# Impact of an Outdoor Smoking Ban at Secondary Schools on Cigarettes, E-Cigarettes and Water Pipe Use among Adolescents: An 18-Month Follow-Up

**DOI:** 10.3390/ijerph15020205

**Published:** 2018-01-25

**Authors:** Andrea D. Rozema, Marieke Hiemstra, Jolanda J. P. Mathijssen, Maria W. J. Jansen, Hans J. A. M. van Oers

**Affiliations:** 1 Department Tranzo, Tilburg School of Social and Behavioral Sciences, Tilburg University, Warandelaan 2, P.O. Box 90153, 5000 LE Tilburg, The Netherlands; marieke_hiemstra@hotmail.com (M.H.); j.j.p.mathijssen@tilburguniversity.edu (J.J.P.M.); hans.van.oers@rivm.nl (H.J.A.M.v.O.); 2 Academic Collaborative Centre for Public Health Limburg, Public Health Service South Limburg (GGD ZL), 6400 AA Geleen, The Netherlands; maria.jansen@ggdzl.nl; 3 Department of Health Services Research, CAPHRI Care and Public Health Research Institute, Maastricht University, 6211 LK Maastricht, The Netherlands; 4 National Institute for Public Health and the Environment (RIVM), 3720 BA Bilthoven, The Netherlands

**Keywords:** school-based intervention, prevention, tobacco control, adolescents, school smoking policies, smoking prevalence, smoke-free, quasi-experimental design, outdoor school ground, secondary schools

## Abstract

The effectiveness of outdoor smoking bans on smoking behavior among adolescents remains inconclusive. This study evaluates the long-term impact of outdoor school ground smoking bans among adolescents at secondary schools on the use of conventional cigarettes, e-cigarettes (with/without nicotine) and water pipes. Outdoor smoking bans at 19 Dutch secondary schools were evaluated using a quasi-experimental design. Data on 7733 adolescents were obtained at baseline, and at 6 and 18-month follow-up. The impact of outdoor smoking bans on ‘ever use of conventional cigarettes’, ‘smoking onset’, ‘ever use of e-cigarette with nicotine’, ‘e-cigarette without nicotine’, and ‘water pipe’ was measured. Multilevel logistic regression analysis was used. At schools with a ban, implementation fidelity was checked. At schools where a ban was implemented, at 18-month follow-up more adolescents had started smoking compared to the control condition. No effect of implementation of the ban was found for smoking prevalence, e-cigarettes with/without nicotine, and water pipe use. Implementation fidelity was sufficient. No long-term effects were found of an outdoor smoking ban, except for smoking onset. The ban might cause a reversal effect when schools encounter difficulties with its enforcement or when adolescents still see others smoking. Additional research is required with a longer follow-up than 18 months.

## 1. Introduction

Smoking continues to be a major cause of illness and death worldwide. In Europe it is one of the main public health problems, since Europe has some of the highest prevalence of tobacco use among adolescents compared to other World Health Organization regions [1]. For example, the average percentage of 15–16 years old European adolescents who have smoked in the last 30 days is 21%, and 10% smokes every day [2]. Because tobacco use mainly starts during adolescence, and because adolescents are extremely sensitive to the addictive substance nicotine [3], protecting youth against the dangers of tobacco use has high priority. 

Alternative tobacco products, i.e., electronic cigarettes with nicotine, electronic cigarettes without nicotine (also known as ‘shisha-pens’) and water pipes (also known as ‘shisha’ or ‘hookah’), have become increasingly popular among adolescents. In fact there is an upward trend in adolescent use of e-cigarettes: rates of ever use have doubled or tripled in recent years [4]. For example, ever use of e-cigarettes use by Dutch adolescents aged 12–16 years is 34% (e-cigarettes with/without nicotine have not been measured separately), which is even higher than the ever use of conventional smoking, i.e., 23%. Use of a water pipe use is also popular, i.e., 23% of the adolescents have used water pipes [5]. 

There are several concerns related to the use of alternative tobacco products. First, these products could undermine years of efforts to ‘denormalize’ smoking [6]. Denormalization of smoking behavior is considered an important population strategy to make non-smoking the generally accepted norm and to prevent deviant behavior, i.e., smoking [7]. Second, there is strong evidence that smoking e-cigarettes/e-cigarettes without nicotine and water pipe act as a ‘stepping stone’ to smoking conventional cigarettes [8,9,10,11,12]. Third, although the level of toxicants is higher in conventional cigarettes, e-cigarette vapors contain toxic substances [13,14] and smoking just one puff of an e-cigarette without nicotine can cause irritation of the airways [15]. Also, water pipe smoking is associated with several negative health outcomes, e.g., increase of heart rate, blood pressure, or even lung cancer and carbon monoxide poisoning [16,17]. In view of these concerns, tobacco control policies aiming to prevent smoking onset should focus not only on conventional cigarettes, but also on alternative tobacco products. 

A possible strategy to prevent or reduce smoking behavior of adolescents is to prohibit smoking at schools, due to the mandatory attendance of adolescents. Whereas, internationally, indoor bans are more or less the norm [18], the importance of implementation of outdoor smoking bans at schools is becoming increasingly apparent [19]. In The Netherlands, until now, 53% of all secondary schools has implemented an outdoor smoking ban [20]. Guidelines established by the Dutch Lung Foundation for an outdoor smoking ban are: (1) the ban should apply to the entire location/site; (2) the ban should apply to everyone (i.e., students, staff and visitors); and (3) the ban should be clearly displayed i.e., by signs and/or in the school regulations [21]. 

Studies on whether or not outdoor smoking bans at secondary schools are effective in reducing smoking behavior remain inconclusive. For example, one study showed the effectiveness on smoking prevalence when schools banned smoking outdoors on the school grounds [22], whereas other studies found no effects of (outdoor) school ground smoking bans [23,24,25,26,27]. Additionally, those studies have methodological shortcomings. For example, most of the studies used a cross-sectional design [23,24,25,26], were performed outside Europe [23,24,25,27], and only one study included a second data point after one year [22]. There is a need for quasi-experimental studies to investigate the impact of an outdoor smoking ban on smoking behavior [28,29].

Given the concerns regarding the use of alternative tobacco products and the conflicting results of studies, the impact of an outdoor smoking ban on both conventional cigarettes *and* alternative tobacco products needs to be investigated using a quasi-experimental design. Therefore, this study aims to evaluate the long-term impact of an outdoor school ground smoking ban at secondary schools (i.e., 18-month follow-up) in a large representative cohort of Dutch adolescents.

## 2. Methods

### 2.1. Procedure and Participants

To evaluate the effect of an outdoor school ground smoking ban on conventional and alternative smoking behavior of adolescents, a quasi-experimental design was used including: (i) schools which decided to implement an outdoor school ground smoking ban (i.e., classified to the experimental condition, *n* = 9) and (ii) schools which did not want to implement an outdoor school ground smoking ban (classified to the control condition, *n* = 10).

For 919 Dutch secondary schools, the smoking policy, and whether or not the schools had the intention to implement an outdoor smoking ban, was known from a national monitor questionnaire [30]. In total, 77 schools were randomly contacted by telephone and asked whether they would participate, taking their current smoking policy into account. Participating schools (*n* = 19) did not differ substantially from the non-participating schools (*n* = 58) in school size (*p* = 0.87), education type (*p* = 0.30) and urbanity (*p* = 0.21). The most common reason for non-participation was lack of time.

**Figure 1:**
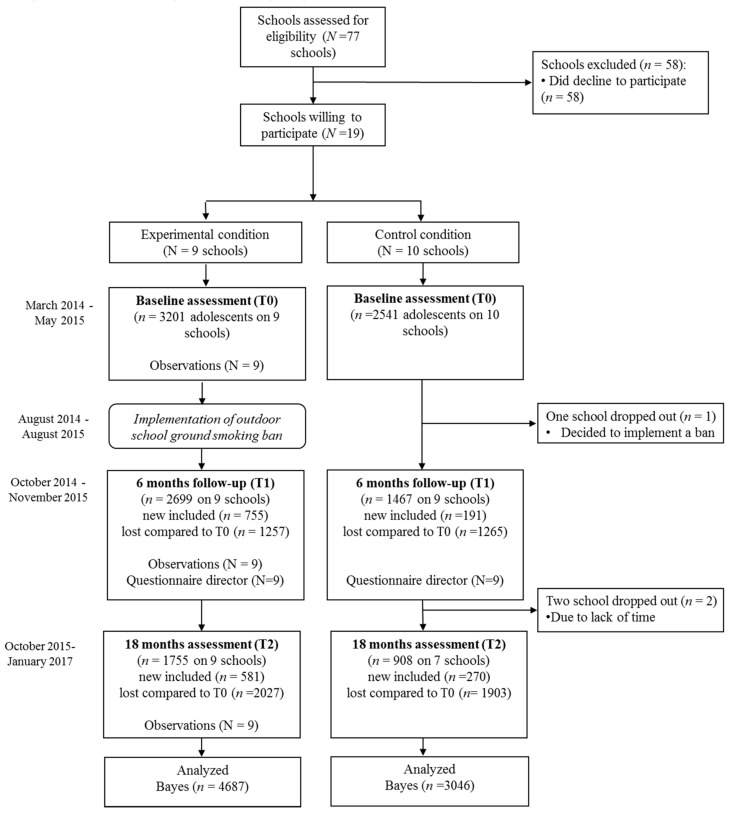
Flow chart showing the inclusion of participants.

Questionnaire data of the adolescents of the participating schools were collected at baseline, and at 6 and 18-month follow-up (T0, T1 and T2, respectively); Figure 1 shows the flow of participants.

At all waves, adolescent questionnaires were administered at school online, or on paper (online: 84% at baseline; 66% at 6 months; 57% at 18 months, and on paper respectively 16%; 34%; 43%). The questionnaires were completed under supervision of a teacher. At 8 schools in the experimental condition, implementation took place between T0 and T1 during the new school year, in August 2014 and in August 2015. One school implemented the ban in February 2015 (after the school vacation). The schools in the control condition only filled out the questionnaires and did *not* implement a school ground smoking ban during the study period. During the study, 3 schools in the control condition dropped out. At baseline 1 control school dropped out, as this school wanted to implement an outdoor smoking ban (*n* = 669). At 18-month follow-up, 2 additional schools in the control condition dropped out due to lack of time (*n* = 472). Moreover, in The Netherlands, because the lowest level of education ends at the 10th grade, students in the 9th grade at T0 had already left school at the 18-month follow-up, i.e., in the experimental condition *n* = 913 students and in the control condition *n* = 450 students dropped out. Furthermore, other absences might be due to dependence on the participation of the teachers, as they had to ask their students to fill out the questionnaire in the classroom.

The study was approved by the Psychological Ethics Committee of Tilburg University (EC-2014.19). Active informed consent was obtained from all participants included in the study. Beforehand, passive informed consent was asked by the parents of the students and they were informed about the research because students were of minority age (<18 years). In total, a small group of parents (*n* = 30) refused study participation of their child at T0. The questionnaire was not administered to these students.

### 2.2. Implementation Fidelity of the Ban

In the experimental condition, it was checked whether the school had actually implemented an outdoor ban. That is, at T1, school directors were asked what their current smoking policy was for conventional smoking and e-cigarettes. At all waves, implementation fidelity was also measured at the individual level, by asking adolescents about the current smoking policy of their school.

To double check implementation fidelity, unnoticed observations were performed at all waves in the experimental condition, by counting the number of smokers (i.e., students and staff) on and off the school grounds during a coffee or lunch break. Additionally, at all waves, it was observed whether or not the school had placed non-smoking signs on the school grounds. Furthermore, in the control condition, it was checked that the school had *not* implemented an outdoor ban, by asking the school directors at T1, and adolescents at all waves, what the current smoking policy of the school was.

### 2.3. Measures

#### 2.3.1. Conventional Cigarettes

The smoking status of conventional cigarettes of adolescents was assessed at each wave. Adolescents were asked to report (using a 5-point scale) which stage of smoking applied to them. Response categories ranged from 1 ‘Never smoked’, 2 ‘Smoked only once or twice’, 3 ‘Smokes occasionally, but not every day’, 4 ‘Smoked in the past/Ex-smokers’, and 5 ‘Smokes every day’. Adolescents who ‘never smoked a cigarette’ were coded as ‘Never user’; and ‘who at least smoked once or twice a cigarette’, ‘who smokes occasionally, but not every day’, ‘who smoked in the past’, and ‘daily smokers’ were coded as ‘Ever user’. If adolescents reported smoking at T0 or T1 they were also defined as ‘ever user’ in the following measurement waves, even though they indicated that they had never smoked (T1:75 and T2:79 of all adolescents). Based on smoking status, we differentiated between *smoking prevalence* and *smoking onset*.

For *smoking prevalence* ‘never users’ were compared with ‘ever users’. For *smoking onset*, only adolescents who were never user at their first measurement (due to absences this could also be T1) were included in the analyses (*n* = 5734) and the development of smoking over time was examined.

#### 2.3.2. Alternative Tobacco Products (E-Cigarettes with/without Nicotine and Water Pipe)

Adolescents were asked the following question ‘How old were you when you used this substance/device for the first time?’, with answer categories ‘I never used this substance/device’, ‘11 years or younger’, ‘12 years’, ‘13 years’, ‘14 years’, ‘15 years’, ‘16 years’, ‘17 years’, ‘18 years’, ‘19 years’ and ‘20 years or older’ for (i) e-cigarettes with nicotine; (ii) e-cigarettes without nicotine (i.e., shisha-pen) and (iii) water pipe. Adolescents saying ‘I never used this substance/device’ were classified as ‘Never user’ and students filled out an age at which they used the substance for the first time were classified as ‘Ever user’.

#### 2.3.3. Sociodemographics

The following sociodemographic variables were included: sex (boys vs. girls), age (in years at T0), migration background (native vs. migrant descent), grade (7th, 8th, 9th and 10th), and education level at T0 (low, average, middle or high). Participants with one or both parents born in a country other than the country of residence were defined as ‘migrant descent’. Furthermore, ‘low education’ refers to schools specialized in students with learning difficulties and pre-vocational secondary education. ‘Average education’ refers to lower general secondary education, ‘middle education’ refers to higher general secondary education, and ‘high education’ refers to pre-university education. In The Netherlands, at primary schools all children are tested and based on this test score, education level of secondary school is determined.

#### 2.3.4. Smoking Policy Variables to Assess Implementation

Current smoking policy of students and staff for conventional cigarettes and for e-cigarettes was measured among the school directors at T1. The questions were ‘It is allowed for students to smoke…’, ‘It is allowed for staff to smoke…’, and ‘It is allowed to smoke e-cigarettes…’, with the answering categories 1 ‘Everywhere on and off the school grounds’, 2 ‘Everywhere on the school grounds’, 3 ‘Only on a specific place on the school grounds’, 4 ‘Just outside the fence off the school grounds’, 5 ‘Only on a specific place off the school grounds’, 6 ‘Everywhere off the school grounds’, 7 ‘Nowhere on and off the school grounds’. An extra answer category was added to the questions regarding the smoking policy of staff and of e-cigarettes, respectively: 8 ‘Only on a specific place in the school building’ and 9 ‘The school does not have a specific policy regarding electronic cigarettes’.

Current smoking policy was also measured by asking all students at all three waves ‘What is the current smoking policy of your school?’ with the answering categories 1 ‘It is allowed to smoke everywhere on the school grounds’, 2 ‘It is only allowed to smoke at a specific place on the school grounds’, 3 ‘It is not allowed to smoke on the school grounds’, 4 ‘The school does not have a school ground’.

### 2.4. Statistical Analysis

Using the SPSS package (SPSS Inc., Chicago, IL, USA), independent sample *t* tests (for continuous outcome variables) and chi-square tests (for categorical outcome variables) were used to compare participants’ characteristics between the experimental and control condition. Attrition analysis to check for differences between participants that dropped out and completers only (i.e., filled out all 3 waves) were conducted using logistic analysis.

Due to the hierarchical structure of the data with 7733 adolescents being nested within 19 schools, and 3 observations per adolescent, a multilevel analysis in Mplus 7.3 (Mplus, Los Angeles, CA, USA) was conducted [31] with three levels (i.e., time, individual, school), to investigate the effect of an outdoor school ground smoking ban on smoking prevalence (‘never use’ vs. ‘ever use’), smoking onset (‘never use’ at first time measurement vs. ‘ever use’ at either T1 or T2), smoking e-cigarette with nicotine, e-cigarette without nicotine and water pipe (‘never use’ vs. ‘ever use’). For the multilevel analyses, the dataset was rearranged from a one-person, one-record dataset (*n* = 7733 person-level dataset) to a one-person, multiple-period dataset (*n_obs_* = 12,571 person-period dataset).

Per outcome variable, a multivariate model was used. Sociodemographic differences (i.e., sex, grade, migration background and education level) between the experimental and control condition were corrected for. Since grade and age are nested, only grade was entered in the model. Also corrected for was the influence of sociodemographics on the development of smoking or alternative tobacco product use over time (i.e., correction for the possible different influence of time on smoking/alternative tobacco products for sex, grade, migration background and education level). To test the school policy effect, the condition variable (experimental/control condition), time (T0, T1, T2), and an interaction between time and condition (time*condition), were entered. Time was added to correct for previous substance use. Random intercepts were added to account for clustered data of individuals within schools, and for repeated measurements within respondent. Variables were centered by subtracting the grand mean or group mean from each variable. Within-subject variables were group mean centered (i.e., time) and between-subject and between-school variables were grand mean centered (i.e., sex, migration background, grade, educational level, and condition).

For categorical three-level analyses, the Bayes estimator was used [32]. We first used the default option in Mplus to converge the model. A second run of a double amount of a fixed number of iterations was used to test the stability of convergence of each model (for specific number of iterations per model see Section 3.1). If a model did not converge by default, the amount of iterations was manually increased until the model converged. Convergence was further verified through diagnostic plots following the recommendations of Muthén [33]. All models showed stable results.

Missing values were handled by Bayes, as Bayes is a full-information estimator [34]. Intra-class correlations (ICCs) on school level were calculated to examine the similarity of adolescents from the same school [35].

## 3. Results

Figure 1 shows the flow of participants. Overall, 5742 adolescents (74%) participated at baseline, 4166 (54%) at T1, and 2663 (34%) at T2. Attrition analysis comparing adolescents that participated at one or two of the waves, compared to adolescents who participated in all three waves (i.e., completers only), showed that girls were more likely to drop out than boys (OR = 1.22, 95% CI = 1.06–1.40, *p* ≤ 0.01). Furthermore, less likely to drop out were migrant adolescents compared to native adolescents (OR = 0.54, 95% CI = 0.44–0.66, *p* ≤ 0.01), smokers compared to non-smokers (OR = 0.77, 95% CI = 0.63–0.94, *p* ≤ 0.01), higher grades compared to lower grades (OR = 0.42, 95% CI = 0.38–0.46, *p* ≤ 0.01), and higher educated adolescents compared to lower educated adolescents (OR = 0.85, 95% CI = 0.79–0.92, *p* ≤ 0.01).

Table 1 shows the characteristics of the adolescents by condition. Significant differences were found between the experimental and control condition for sex, age, grade, educational level and migration background. Therefore, further analyses were controlled for these sociodemographic differences between both conditions. Table 2 shows the number (and percentages) of ever users for smoking onset, smoking prevalence, e-cigarette use (with/without nicotine) and water pipe use.

### 3.1. Multilevel Analysis

Table 3 and Table 4 present the results of the implementation of outdoor school ground smoking bans on smoking prevalence, smoking onset, and use of alternative tobacco products. Significant results of sociodemographic on smoking prevalence, e-cigarette use with/without nicotine and water pipe use were in line with expectations: girls used less than boys, other ethnicity than Dutch used more than Dutch adolescents, higher grades used more than lower grades and higher education used less than lower education levels. For smoking prevalence, smoking onset and e-cigarettes with nicotine an increase over time was found. For implementation of school smoking policies only significant results were found for smoking onset (time*condition). This means that students at schools which implemented outdoor school ground smoking bans, had a higher probability to start smoking over time compared to the control condition (β = 1.42, 95% BCI * = 0.21–2.96; * Bayesian Credibility Interval). For smoking prevalence and e-cigarettes with/without nicotine, and water pipe use, no interaction effects (time*condition) were found.

Variance components showed that the variation between individuals and schools was significant in all models. Variance for the intercept and slope of individual level indicated a variation between individuals in initial use and use over time. For school level, variation between the schools in initial use and use over time developed differently. The Intra-class correlations (ICCs) to determine the effects of school clustering were between 0.06 and 0.16, indicating that 6–16% of the variance could be explained by a school effect (Table 3 and Table 4).

### 3.2. Implementation Fidelity of the Ban

On school level, all school directors in the experimental condition reported that they had implemented the ban (*n* = 9), and in the control condition they confirmed that they had not implemented the ban (*n* = 9). In the experimental condition, schools differed in their implementation form, i.e., 5 schools implemented the ban on the school grounds only for students, and 4 schools implemented the ban on the school grounds for both students and staff. One school in the experimental condition reported that they did not have a specific policy regarding e-cigarettes, the remaining 8 schools reported that the outdoor smoking ban also applied to e-cigarettes.

Implementation fidelity was also measured at individual level, by asking the adolescents about the current smoking policy of their school at all waves. At baseline, in both the experimental (4.3%) and control condition (5.8%), only a small percentage of the students reported that it was prohibited to smoke on the school grounds. After implementation (6-month follow-up) this had increased to 81.6% in the experimental condition vs. 7.4% in the control condition. At 18-month follow-up, the results were similar to those found at T1: 72.7% of the students reported that it was prohibited to smoke on the school grounds in the experimental condition vs. 10.2% in the control condition.

The observations in the experimental condition demonstrated that, at baseline, at 8 of the 9 schools the adolescents smoked on the school grounds (89%). After implementation (T1), no smoking adolescents were observed at all 9 school grounds (0%). At 18-month follow-up, at 2 schools (22%) smoking behavior was observed on the school grounds (i.e., at one school smoking students were observed, and at the other school both smoking students and staff were observed). In contrast, students smoking cigarettes were more often seen off the school grounds after implementation: at T0 at 4 schools smoking students were seen off the school grounds (44%) vs. 8 at T1 (89%) and 7 at T2 (78%). Furthermore, 4 schools (44%) placed signs at their school grounds at T1 and this had increased to 5 schools (56%) at T2.

## 4. Discussion

This is the first study to evaluate the long-term impact of an outdoor school ground smoking ban at secondary schools on the use of conventional cigarettes and of alternative tobacco products among adolescents. Results show that schools with and without an outdoor ban at 18-month follow-up did not significantly differ on smoking prevalence, use of e-cigarettes (with/without nicotine) and water pipe among adolescents. However, except for smoking onset, a significant effect was found. At the schools that had implemented outdoor school ground smoking bans, more adolescents started smoking.

The international evidence regarding the effectiveness of outdoor smoking bans remains equivocal and, in line with another study, an outdoor smoking ban might not always protect adolescents from taking up smoking [27]. Nevertheless, whether or not school smoking policies are effective, depends on whether: (i) the policy is strictly enforced and sanctioned; (ii) adolescents do not feel the pressure to smoke; and (iii) anti-smoking beliefs are internalized so that non-smoking becomes the generally accepted norm [36]. All these points are important when interpreting the results of the present study.

First, although all schools in the experimental condition implemented the ban, the schools might have encountered difficulties with enforcement or differentiated in the level of enforcement (e.g., strict vs. non-strict), which might explain the absence of positive results (i.e., a reduction in smoking behavior). Also, enforcement is challenging after implementation of a smoking ban and adherence is often far from optimal [37,38,39]. During adolescence, a ban (which is a forced choice) might threaten self-autonomy and can backfire, and might even exacerbate deviant behavior, e.g., smoking [40]. Jancey et al. [40] also reported other reasons for noncompliance of smoking bans: e.g., reluctance to leave the school grounds, smoking necessity, unintentional noncompliance through confusion of school boundaries, or avoiding detection (e.g., going to places where one is unlikely to be observed). In general, future research using a quasi-experimental design should also focus on the enforcement and sanctions related to bans at schools.

Second, adolescents still saw others smoking during school hours, since most schools in the experimental condition tolerated smoking off the school grounds. As a result, adolescents might have felt pressure to conform to other smoking behavior [36]. Actual smoking might have shifted to, for example, off the school grounds. Observational data of the present study and our previous study support this: i.e., more smoking students were observed near the school grounds after implementation [41]. Additionally, at baseline in both conditions, 40% of the adolescents reported that they smoked outside the school grounds; however, this percentage increased to 47% in the control condition, and to 57% in the experimental condition at 18-month follow-up. In fact, the only study which showed the effectiveness of outdoor smoking bans concerned schools that prohibited smoking on the school grounds *and* in the immediate surroundings of the school [22]. School tobacco policies might cause a reversal effect when adolescents find easily accessible alternative locations to smoke off the school grounds [36], and when the anti-normative behavior of adolescents is salient at the entrance of the school.

Finally, although implementation of smoke-free environments (such as outdoor school ground smoking bans) contributes to non-smoking as the generally accepted norm [19] and to reduced exposure to second-hand smoke [42], 18 months might be too short a period to find positive results in behavior change. Adolescents might not yet have internalized anti-smoking beliefs. Changing a norm is a gradual process that takes time [43]. For example, in The Netherlands, after implementation of the smoking ban in hospitality venues, there was much resistance and compliance/acceptance was only about 50% in 2010 and increased to 90% only after 5 years [44]. Therefore, a follow-up period longer than 18 months is needed, as the impact of outdoor smoking bans might have a delayed effect. Future studies could compare the control schools with schools that have had an outdoor smoking ban for a considerable number of years, e.g., ≥5 years. Additionally, school using a bottom-up approach during implementation (i.e., consulting key stakeholders), should compared with schools using a top-down approach (i.e., no consultation of key stakeholders), as this might increase support for the ban and consequently might influence impact of the ban.

Schools might consider to prohibit smoking anywhere on the school grounds, and apply the ban to all types of tobacco products (including alternative tobacco products), thereby strengthening non-smoking as the norm. An integral approach, i.e., integration of outdoor smoking bans in different environments, might be a precondition to more effectively influence the non-smoking norm. For example, to be effective, smoking bans should be implemented not only at schools, but also at other places such as at adolescents’ home [45], playgrounds [46], or other locations where adolescents spend regular time, e.g., sport grounds or beaches/public swimming pools. Indeed, it is reported that smoking norms at home seem to predict the effect of school smoking restrictions [47]. Moreover, bans should be extended with other interventions/policies often found to be effective in reducing adolescents’ smoking, such as school programs focusing on social influences and competence [48], or other effective tobacco control policies aiming to reduce smoking behavior among adolescents, e.g., increasing price [49].

## 5. Strengths and Limitations

A strength of this study is its quasi-experimental design. Moreover, the effectiveness of outdoor smoking bans on the use of alternative tobacco products among adolescents was investigated for the first time; this is valuable due to the clear link between alternative tobacco products and the uptake of conventional cigarettes [8,9,10,11,12]. Additionally, few studies have distinguished between e-cigarettes with nicotine and without nicotine, which is indicated as a research priority [4].

The present study also has some limitations. First, the schools were not randomized and we had therefore no influence on which school implemented the smoking policy and which did not. This leaded to differences in sociodemographics (i.e., sex, grade, migration background and education level) between conditions. However, we controlled for differences in sociodemographics between the experimental and control condition. Second, although there was a substantial amount of missing data, these absences were probably random, since the prevalence rates of tobacco products were similar to those of a representative Dutch study among adolescents [5]. Third, self-reported data were used, increasing the risk of underreporting of smoking [50]. Nevertheless, confidentiality was assured during the study, which is effective in increasing the reliability of self-reported data of smoking behavior [51]. Fourth, the schools differed somewhat in implementation forms; however, there was insufficient power to divide the experimental condition into different groups. Internationally, more research including more schools is needed to investigate different guidelines/definitions of outdoor smoking bans and which implementation form is the best.

## 6. Conclusions

An outdoor smoking ban did not appear to affect smoking prevalence and use of e-cigarettes (with/without nicotine) and water pipe use after 18 months. Except for smoking onset, a significant effect was found. At the schools that had implemented outdoor school ground smoking bans, more adolescents started smoking. To prevent relocation of smoking, schools might strictly enforce the ban and apply the ban not only on school grounds but also in the immediate surrounding off the school grounds; or schools might aim to prevent students leaving the school grounds during school hours. Additional long-term studies are required to determine the longer-term effects on both conventional smoking and use of alternative tobacco products, taking into account the different implementation forms. Overall, schools might consider to prohibit smoking anywhere on the school grounds, and apply the ban to all kind of tobacco products, thereby strengthening non-smoking as the norm and decreasing exposure to second-hand smoke.

## Figures and Tables

**Table 1:** Characteristics of adolescents in the experimental and control condition #.

		Experimental Condition (E) (*n* = 4687)	Control Condition (C) (*n* = 3046)	Significant Difference between E and C ^+^
Sex				χ^2^ = 10.985 (1), *p* = 0.001
	Boy	2355 (50.7)	1647 (54.5)	
	Girl	2293 (49.3)	1373 (45.5)	
Migration background				χ^2^ = 40.53 (1), *p* < 0.001
	Native	3815 (83.5)	2317 (77.6)	
	Migrant descent ^!^	755 (16.5)	668 (22.4)	
Age in years (mean (SD)		13.9 (1.06)	13.5 (1.10)	*t*(5623) = −15.763, *p* < 0.001
Education level *				χ^2^ = 1087.36 (3), *p* < 0.001
	Low	1557 (49.2)	283 (11.3)	
	Average	979 (31.0)	891 (35.7)	
	Middle	297 (9.4)	763 (30.6)	
	High	329 (10.4)	559 (22.4)	
Grade				χ^2^ = 172.47 (5), *p* < 0.001
	7th	1003 (21.5)	839 (27.7)	
	8th	1351 (29.0)	933 (30.8)	
	9th	1707 (36.6)	1116 (36.8)	
	10th	566 (12.1)	118 (3.9)	
	11th	12 (0.3)	8 (0.3)	
	12th	21 (0.5)	18 (0.6)	

# Values are numbers (percentages) unless stated otherwise. ^+^ χ^2^ categorical; *t*-test continuous. * Education: low education refers to schools specialized in students with learning difficulties and pre-vocational secondary education, average education refers to lower general secondary education, middle education refers to higher general secondary education, and high education refers to pre-university education. ^!^ Migrant descent = one or both parents born in a country other than The Netherlands.

**Table 2:** Smoking behavior (% of ever users) at baseline (T0), and at 6-month (T1) and 18-month follow-up (T2) measurements.

		Total Sample	Experimental Condition ‘Ever Users’	Control Condition ‘Ever Users’	Total ‘Ever Users’
Smoking prevalence		n	*n* (%)	*n* (%)	*n* (%)
	T0	5695	745 (23.4)	485 (19.3)	1230 (21.6)
	T1	4690	1120 (37.6)	655 (38.2)	1775 (37.8)
	T2	4020	1547 (59.6)	771 (54.2)	2318 (57.7)
Smoking onset					
	T0	4465	-	-	-
	T1	2347	132 (6.7)	84 (7.4)	216 (7.0)
	T2	1473	279 (21.0)	122 (15.8)	401 (19.1)
E-cigarette with nicotine					
	T0	5517	481 (15.6)	260 (10.7)	741 (13.4)
	T1	4069	402 (15.3)	208 (14.5)	610 (15.0)
	T2	2568	346 (20.5)	142 (16.1)	488 (16.3)
E-cigarette without nicotine					
	T0	5514	921 (29.8)	654 (26.9)	1575 (28.6)
	T1	4061	846 (32.2)	414 (28.9)	1260 (31.0)
	T2	2559	580 (34.5)	251 (28.6)	831 (32.5)
Water pipe					
	T0	5517	725 (23.4)	455 (18.8)	1180 (21.4)
	T1	4066	673 (25.6)	266 (18.5)	939 (23.1)
	T2	2561	560 (33.3)	191 (21.7)	751 (29.3)

**Table 3:** Multivariate Bayes analyses of the implementation of the outdoor school ground smoking ban on ‘ever use’ of *smoking prevalence* (*n* = 5546) and *smoking onset* (*n* = 4365) controlling for sex, migration background, grade and education level.

		Smoking Prevalence			Smoking Onset		
		Median Estimate (Posterior SD) ^+^	One-Tailed *p*-Value	Bayesian 95% Credibility Interval *	Median Estimate (Posterior SD) ^+^	One-Tailed *p*-Value	Bayesian 95% Credibility Interval *
Regression							
coefficients	Sex	**−2.56 (0.70)**	**0.000**	−4.05–−1.27 *	−0.06 (0.41)	0.44	−0.092–0.74
	Migration background	**2.24 (0.91)**	**0.005**	0.54–4.11 *	−0.66 (0.63)	0.14	−1.95–0.53
	Grade	**5.89 (0.72)**	**0.000**	4.69–7.48 *	0.048 (0.26)	0.43	−0.49–0.054
	Educational level	**−4.20 (0.71)**	**0.000**	−5.77–−3.00 *	−0.20 (0.33)	0.26	−0.90–0.42
	Time	**3.64 (1.15)**	**0.000**	1.85–6.24 *	**2.90 (1.26)**	**0.000**	1.00–5.82 *
	Condition	−2.80 (1.75)	0.06	−6.22–0.68	−1.24 (1.31)	0.15	−4.18–0.99
	Condition*time	0.52 (0.57)	0.17	−0.58–1.66	**1.42 (0.70)**	**0.01**	0.21–2.96 *
Variance	Intercept variance individual level	**331.74 (86.68)**	**0.000**	213.79–547.02 *	**39.15 (13.09)**	**0.000**	17.40–69.44 *
components	Slope variance individual level	**0.14 (0.20)**	**0.000**	0.004–0.75 *	**0.15 (0.23)**	**0.000**	0.01–0.83 *
	Intercept variance school level	**41.39 (34.91)**	**0.000**	15.50–143.52 *	**7.31 (8.11)**	**0.000**	2.09–30.38 *
	Slope variance school level	**1.26 (1.03)**	**0.000**	0.44–4.19 *	**0.47 (0.94)**	**0.000**	0.11–3.28 *
	Covariance between school level intercept and slope	**5.15 (5.12)**	**0.005**	1.03–19.92 *	−1.08 (2.48)	0.17	−7.94–1.21
Fit index	School ICC	0.11			0.16		
	Iterations ^~^	419,300			757,600		

Note. Sex: 1 = boy, 2 = girl, Migration background: 0 = Dutch, 1 = other, Condition: 0 = control, 1 = experimental, Time: 1, 2, 3; * = significant 2-tailed *p*-value (significant = bold), ICC = Intra-class correlations, ^+^ Probit coefficient, ^~^ Model converged if Potential Scale Reduction (PSR) value was below 1.1.

**Table 4:** Multivariate Bayes analyses of the implementation of the outdoor school ground smoking ban on ‘ever use’ of *e-cigarettes with nicotine* (*n* = 5407), *e-cigarettes without nicotine* (*n* = 5404), and *water pipe use* (*n* = 5405) controlling for sex, migration background, grade and education level.

		E-Cigarettes with Nicotine			E-Cigarettes without Nicotine			Water Pipe		
		Median Estimate (Posterior SD) ^+^	One-Tailed *p*-Value	Bayesian 95% Credibility Interval *	Median Estimate (Posterior SD) ^+^	One-Tailed *p*-Value	Bayesian 95% Credibility Interval *	Median Estimate (Posterior SD) ^+^	One-Tailed *p*-Value	Bayesian 95% Credibility Interval *
Regression										
coefficients	Sex	**−0.93 (0.12)**	**0.000**	−1.17–−0.700 *	**−0.94 (0.09)**	**0.000**	−1.11–−0.7700 *	**−0.93 (0.03)**	**0.000**	−1.19–−0.69 *
	Migration Background	**0.33 (0.14)**	**0.007**	0.07–0.600 *	**0.27 (0.13)**	**0.02**	0.02–0.5100 *	**0.75 (0.17)**	**0.000**	0.43–1.11 *
	Grade	**0.66 (0.08)**	**0.000**	0.51–0.820 *	**0.48 (0.06)**	**0.000**	0.37–0.6100 *	**1.15 (0.11)**	**0.000**	0.94–1.36 *
	Educational level	**−0.036 (0.08)**	**0.000**	−0.51–−0.210 *	**−0.45 (0.06)**	**0.000**	−0.57–−0.3300 *	**−0.57 (0.10)**	**0.000**	−0.077–−0.40 *
	Time	**1.09 (0.26)**	**0.000**	0.59–1.610 *	0.40 (0.24)	0.05	−0.06–0.89	0.20 (0.25)	0.21	−0.31–0.70
	Condition	−0.19 (0.41)	0.31	−1.01–0.59	−0.03 (0.32)	0.46	−0.67–0.59	0.32 (0.39)	0.20	−0.43–1.10
	Condition*time	−0.42 (0.23)	0.03	−0.88–0.02	−0.15 (0.20)	0.24	−0.55–0.26	0.20 (0.25)	0.21	−0.30–0.69
Variance	Intercept variance individual level	**5.42 (0.83)**	**0.000**	3.77–6.810 *	**5.34 (0.54)**	**0.000**	4.43–6.6100 *	**9.08 (1.41)**	**0.000**	6.52–11.98 *
components	Slope variance individual level	**0.49 (0.17)**	**0.000**	0.24–0.890 *	**0.44 (0.12)**	**0.000**	0.20–0.6700 *	**0.58 (0.22)**	**0.000**	0.19–1.04 *
	Intercept variance school level	**0.64 (0.31)**	**0.000**	0.31–1.480 *	**0.37 (0.19)**	**0.000**	0.17–0.8800 *	**0.55 (0.29)**	**0.000**	0.25–1.36 *
	Slope variance school level	**0.13 (0.07)**	**0.000**	0.06–0.320 *	**0.12 (0.06)**	**0.000**	0.06–0.2700 *	**0.15 (0.08)**	**0.000**	0.07–0.38 *
	Covariance between school level intercept and slope	0.002 (0.12)	0.49	−0.23–0.25	0.004 (0.07)	0.47	−0.13–0.15	−0.04 (0.11)	0.33	−0.29–0.15
Fit index	School ICC	0.11			0.06			0.06		
	Iterations ^~^	13,000			5700			4800		

Note. Sex: 1 = boy, 2 = girl, Migration background: 0 = Dutch, 1 = other, Condition: 0 = control, 1 = experimental, Time: 1, 2, 3; * = significant 2 tailed *p*-value (significant = bold), ICC = Intra-class correlations, ^+^ Probit coefficient, ^~^ Model converged if Potential Scale Reduction (PSR) value was below 1.1.
